# DADA: Degree-Aware Algorithms for Network-Based Disease Gene Prioritization

**DOI:** 10.1186/1756-0381-4-19

**Published:** 2011-06-24

**Authors:** Sinan Erten, Gurkan Bebek, Rob M Ewing, Mehmet Koyutürk

**Affiliations:** 1 Department of Electrical Engineering and Computer Science, Case Western Reserve University, Cleveland, OH, USA; 2 Case Center for Proteomics and Bioinformatics, Case Western Reserve University, Cleveland, OH, USA; 3 Case Comprehensive Cancer Center, Case Western Reserve University, Cleveland, OH, USA; 4 Genomic Medicine Institute, Cleveland Clinic, Cleveland, OH, USA; 5 Department of Genetics, Case Western Reserve University, Cleveland, OH, USA

## Abstract

**Background:**

High-throughput molecular interaction data have been used effectively to prioritize candidate genes that are linked to a disease, based on the observation that the products of genes associated with similar diseases are likely to interact with each other heavily in a network of protein-protein interactions (PPIs). An important challenge for these applications, however, is the incomplete and noisy nature of PPI data. Information flow based methods alleviate these problems to a certain extent, by considering indirect interactions and multiplicity of paths.

**Results:**

We demonstrate that existing methods are likely to favor highly connected genes, making prioritization sensitive to the skewed degree distribution of PPI networks, as well as ascertainment bias in available interaction and disease association data. Motivated by this observation, we propose several statistical adjustment methods to account for the degree distribution of known disease and candidate genes, using a PPI network with associated confidence scores for interactions. We show that the proposed methods can detect loosely connected disease genes that are missed by existing approaches, however, this improvement might come at the price of more false negatives for highly connected genes. Consequently, we develop a suite called DADA, which includes different uniform prioritization methods that effectively integrate existing approaches with the proposed statistical adjustment strategies. Comprehensive experimental results on the Online Mendelian Inheritance in Man (OMIM) database show that DADA outperforms existing methods in prioritizing candidate disease genes.

**Conclusions:**

These results demonstrate the importance of employing accurate statistical models and associated adjustment methods in network-based disease gene prioritization, as well as other network-based functional inference applications. DADA is implemented in Matlab and is freely available at http://compbio.case.edu/dada/.

## Introduction

Identification of disease-associated genes is an important step toward enhancing our understanding of the cellular mechanisms that drive human diseases, with profound applications in modeling, diagnosis, prognosis, and therapeutic intervention [1]. Genome-wide linkage and association studies (GWAS) in healthy and affected populations identify chromosomal regions potentially containing hundreds of candidate genes possibly associated with genetic diseases [2]. Investigation of these candidates using experimental methods is an expensive task, thus not always a feasible option. Consequently, computational methods play an important role in prioritization and identification of the most likely disease-associated genes by utilizing a variety of data sources such as gene expression [3,4], functional annotations [4-7], and protein-protein interactions (PPIs) [3,8-14]. The scope of methods that rely on functional annotations is limited because only a small fraction of genes in the genome are currently annotated.

In recent years, several algorithms have been proposed to incorporate topological properties of PPI networks in understanding genetic diseases [3,8,13]. These algorithms mostly focus on prioritization of candidate genes and mainly exploit the notion that the products of genes associated with similar diseases have a higher chance of being connected in the network of PPIs. However, an important challenge for these applications is the incomplete and noisy nature of the PPI data [15]. Missing interactions and false positives affect the accuracy of methods based on local network information such as direct interactions and shortest distances. Few global methods based on simulation of information flow in the network (*e.g*., random walks [8,13] or network propagation [14]) get around this problem to a certain extent by considering multiple alternate paths and whole topology of PPI networks. Nevertheless, as we demonstrate in this paper, these methods favor genes whose products are highly connected in the network and perform poorly in identifying loosely connected disease genes.

In this study, we propose novel statistical adjustment methods to correct for degree bias in information flow based disease gene prioritization. These methods aim to assess the statistical significance of the network connectivity of a candidate gene to known disease genes. For this purpose, we use three reference models that take into account the degree distribution of the PPI network: (*i*) reference model based on degree distribution of known disease gene products, (*ii*) reference model based on the degree of candidate gene products, and (*iii*) likelihood ratio test using eigenvector centrality as the reference model.

We present comprehensive experimental results demonstrating that the proposed statistical adjustment methods are very effective in detecting loosely connected disease genes which are generally less studied, thus potentially more interesting in terms of generating novel biological knowledge. However, we observe that these methods might perform less favorably in identifying highly connected disease genes. Consequently, we develop three uniform prioritization methods that effectively integrate existing algorithms with the proposed statistical adjustment methods, with a view of delivering high accuracy irrespective of the network connectivity of target disease genes. These methods choose the measure to rank candidate genes (raw scores vs. statistically adjusted scores), based on several criteria that take into account the network degree of candidates. Finally, we present comprehensive experimental results in the Results section. These results show that the resulting prioritization methods, implemented in Matlab as a suite called DADA, outperform existing approaches in identifying disease-associated genes.

## Background

In this section, we introduce the network-based disease gene prioritization problem in a formal framework. We then discuss two information flow based disease gene prioritization algorithms that represent the state of the art in network-based disease gene prioritization. These algorithms are random walk with restarts and network propagation. Next, we present detailed experimental results to demonstrate the limitations of these algorithms in the context of the degree distribution of PPI networks and ascertainment bias in interaction data. Based on these observations, we motivate our approach of applying statistical adjustment to the scores computed by these algorithms.

### Network-based candidate disease gene prioritization

There exists a wide range of disease gene prioritization methods that are based on the analysis of the topological properties of PPI networks. These methods commonly rely on the observation that the products of genes that are associated with similar diseases have a higher likelihood of physically interacting [11]. It is important to note here the distinction between genes and their products. Genome-wide association studies focus on identifying *genes *that are associated with a disease of interest. Network-based prioritization aims to aid this effort by inferring functional associations between genes based on the interactions among their products, *i.e*., *proteins*. For this reason, any reference to interactions between genes in this paper refers to the interactions between their products.

Existing methods for network-based disease gene prioritization can be classified into two main categories; (i) localized methods, *i.e*., methods based on direct interactions and shortest paths between known disease genes and candidate genes [3,9,16], (ii) global methods, *i.e*., methods that model the information flow in the cell to assess the proximity and connectivity between known disease genes and candidate genes. Several studies show that global approaches, such as random walk and network propagation, clearly outperform local approaches [13,14,17]. For this reason, we focus on global methods in this paper.

For a given disease of interest *D*, the input to the disease gene prioritization problem consists of two sets of genes, the seed set  and the candidate set . The *seed set * specifies prior knowledge on the disease, *i.e*., it is the set of genes known to be associated with *D *and diseases similar to *D*. Each gene  is also associated with a similarity score *σ*(*v, D*), indicating the known degree of association between *v *and *D*. The similarity score for gene *v *is computed as the maximum phenotypic similarity between *D *and any other disease associated with *v*, based on clinical description of diseases (a detailed discussion on computation of phenotypic similarity scores can be found in the Methods section below). The *candidate set * specifies the genes, one or more of which are potentially associated with disease *D *(e.g., these genes might lie within a linkage interval that is identified by association studies). The overall objective of network based disease prioritization is to use a human PPI network , to compute a score *α*(*v, D*) for each gene , such that *α*(*v, D*) represents the likelihood of *v *to be associated with *D*.

The PPI network  consists of a set of gene products  and a set of undirected interactions  between these gene products, where  represents an interaction between  and . Since PPI data might be obtained from various resources, interactions are often assigned confidence scores indicating their reliability. In other words, the network is also associated with a function , such that *w*(*uv*) indicates the reliability of interaction . Finally, the set of interacting partners of a gene product  is defined as  and the total reliability of known interactions of *v *is defined as  which we refer as *weighted degree *throughout this paper. Global prioritization methods use this network information to compute *α *by propagating *σ *over . Candidate proteins are then ranked according to *α *and novel genes that are potentially associated with the disease of interest are identified based on this ranking.

### Random walk with restarts

This method simulates a random walk on the network to compute the proximity between two nodes by exploiting the global structure of the network [18,19]. It is used in a wide range of applications, including identification of functional modules [20] and modeling the evolution of social networks [21]. Recently, random walk with restarts has also been applied to candidate disease gene prioritization [8,13].

In the context of disease gene prioritization, random walk with restarts is applied as follows. A random walk starts at one of the proteins in . At each step, the random walk either moves to a randomly chosen neighbor  of the current protein *v *or it restarts at one of the proteins in the seed set . The probability of restarting at a given time step is a fixed parameter denoted by *r*. For each restart, the probability of restarting at  is a function of *σ*(*v, D*), *i.e*., the degree of association between *v *and the disease of interest. For each move, the probability of moving to interacting partner *u *of the current protein *v *is proportional to the reliability of the interaction between *u *and *v*, *i.e*., *w*(*uv*). After a sufficiently long time, the probability of being at node *v *at a random time step provides a measure of the functional association between *v *and the genes known to be associated with *D *[8,13]. Algorithmically, random-walk based association scores can be computed iteratively as follows:(1)

Here, *ρ *denotes the restart vector with  for  and 0 otherwise. *P*_RW _denotes the stochastic matrix derived from , *i.e*., *P*_RW_(*u, v*) = *w*(*uv*)*/W*(*v*) for  and 0 otherwise. For each , *x_t_*(*v*) denotes the probability that the random walk will be at *v *at time *t*, where *x*_0 _= *ρ*. For each gene *v*, the resulting random-walk based association score is defined as *α*_RW_(*v, D*) = lim*_t→∞ _x_t_*(*v*). The elements in the resulting vector *α *represent the proximity of each protein to the proteins in the seed set.

### Network propagation

Propagation based models have been previously shown to be effective in network based functional annotation of proteins [22]. In recent work, Vanunu *et al*. [14] propose a network propagation algorithm to compute the association between candidate proteins and known disease genes. They define a prioritization function which models an information pump that originates at the seed sets. This idea is very similar to that of random walk with restarts, with one key difference. Namely, in network propagation, the flow of information is normalized by not only the total outgoing flow from each node, but also the total incoming flow into each node.

In other words, the matrix *P*_RW _is replaced by a matrix *P*_NP_, in which each entry is normalized with respect to row and column sums. The resulting propagation based model can also be simulated iteratively as follows:(2)

Here, the propagation matrix *P*_NP _is computed as  for , 0 otherwise. For each , *y_t_*(*v*) denotes the amount of disease association information at node *v *at step *t*, where *y*_0 _= *ρ*. For each gene *v*, the resulting network propagation based association score is defined as *α*_NP_(*v, D*) = lim*_t→∞ _y_t_*(*v*). In this model, 0 *≤ r ≤ *1 is also a user-defined parameter that is used to adjust the relative importance of prior knowledge and network topology.

### Role of network degree

**Figure 1:**
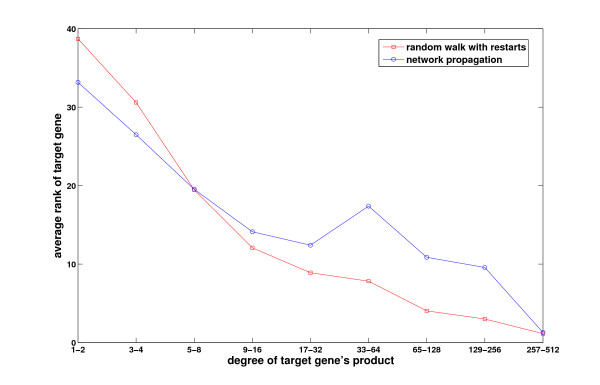
**The effect of connectivity of the target gene on the performance of existing methods**. The performance of existing information flow based methods depends on the number of known interactions of the true disease gene. x-axis represents number of interactions, y-axis represents the average rank of true disease genes with the corresponding degree.

**Figure 2:**
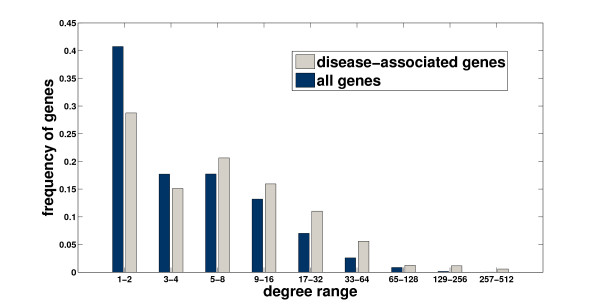
**Histogram of the number of interactions of disease genes and all genes in the network**.

In order to motivate our approach, we evaluate here the performance of random walk with restarts and network propagation with respect to the network degree (number of known interactions) of candidate genes. As shown in Figure 1, these methods are clearly biased toward scoring highly connected proteins higher. In this figure, the performance measure is the average rank of the true candidate protein among other 99 proteins in the same linkage interval (please see the Methods section for a description of the experimental set-up used to generate these results). As evident in the figure, existing global methods work very well in predicting highly connected proteins, whereas they perform quite poorly for loosely connected proteins, especially for those with degree less than 6. Furthermore, as seen in Figure 2, the degree distribution of known disease genes is slightly biased toward highly connected genes, however there exist many disease genes that are loosely connected as well. For this reason, it is at least as important to correctly identify loosely connected disease genes as to identify those that are highly connected, in order to remove the effect of ascertainment bias in PPI data and known disease associations. In this context, the term ascertainment bias refers to the distortion in the degree distribution of PPI networks that might result from the difference in how different proteins are assayed for interactions. In particular, it is expected that highly studied proteins in the literature generally have more known interactions and disease associations compared to those that are less studied.

The dependency of performance on network degree can be understood by carefully inspecting the formulation of random walk and network propagation models. Random walk with restarts is actually a generalization of Google's well-known page-rank algorithm [23]. Indeed, for *r *= 0, *α *is solely a measure of network centrality. Therefore, for any *r >*0, *α*(*v, D*) contains a component that represents the network centrality of *v*, in addition to its association with *D*. Network propagation alleviates this problem by normalizing the incoming flow into a gene, therefore provides a slightly more balanced performance compared to random walk with restarts. However, as evident in the figure, its performance is still influenced heavily by node degrees. Motivated by these insights, we argue that the association scores computed by these algorithms have to be statistically adjusted with respect to reference models that take into account the degree distribution of the network.

## Methods

In this section, we propose several reference models for assessing the significance of network-based disease association scores. Subsequently, we discuss how these models can be used in conjunction with existing methods to obtain uniform prioritization methods that can deliver high accuracy regardless of the centrality of candidate genes. Next, we introduce the disease and PPI datasets and the details of the experimental settings used.

### Reference Models for Statistical Adjustment

Here, we consider three different reference models for assessing the significance of disease association scores obtained by an information flow based prioritization algorithm: (i) a model that compares the association scores of candidate genes computed using the original seed set with an empirical distribution that is obtained by using randomly generated seed sets that match the degree distribution of the original seed set, (ii) a model that compares the association scores of candidate genes to an empirical distribution that is derived from other genes with similar degree in the network, using a fixed seed set, (iii) a model that assesses the likelihood-ratio of the association of a gene with the seed set with respect to its network centrality. Here, for the sake of clarity, we describe each model assuming that random walk based restarts is used to compute raw association scores, however, the methods are directly applicable to network propagation as well (we also drop the subscript RW from our notation for simplicity).

### Reference model based on seed degrees

The objective here is to generate a reference model that captures the degree distribution of seed proteins accurately. To this end, we compare the association score *α*(*v, D*) for each protein with scores computed using random seed sets (by preserving the degree distribution of the seed genes). The expectation here is that false positives that correspond to centralized and highly connected proteins will have high association scores even with respect to these randomly generated seed sets. Such an observation implies that their observed association with the actual seed set is most likely artificial, thus the statistical significance of their association score is low. On the other hand, loosely connected proteins that lie close to the seed proteins will be assigned high significance although their association scores are generally lower than that of highly connected proteins.

Given a disease *D*, seed set , and candidate set , this reference model is implemented as follows:

• We first compute network-based association scores *α*(*v, D*) for the original seed set , using the procedure described by Equation 1.

• Then, based on the original seed set , we generate a random instance  that represents  in terms of weighted degree distribution.  is generated as follows:

- First, a bucket  is created for each protein .

- Then, each protein  is assigned to bucket  if *W *(*v*) *− W *(*u*) *< W *(*v*) *− W *(*u'*) for all , where ties are broken randomly.

- Subsequently,  is generated by choosing a protein from each bucket uniformly at random. It can be observed that each protein in  is represented by exactly one protein in , thus the total weighted degree of proteins in  is expected to be very close to that of .

• For 1 *≤ i ≤ n*, the association scores *α*^(*i*) ^for seed set  are computed using Equation 1. Here, *n *is a sufficiently large number that is used to obtain a representative sampling {*α*^(1)^, *α*^(2)^, *α*^(3)^, ..., *α*^(*n*)^} of the population of association scores for seed sets that match the size and degree distribution of  (we use = 1000 in our experiments).

• We then estimate the mean of this distribution as  and the standard deviation as .

• Finally, we compute the adjusted score for each gene *v *as .

**Figure 3:**
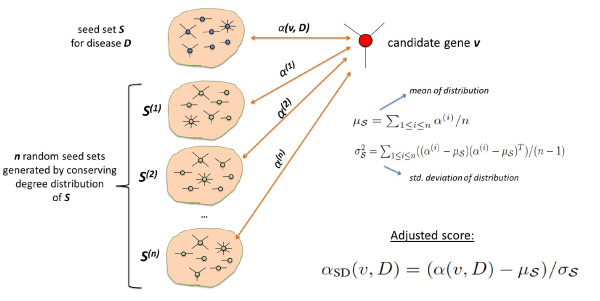
**Statistical adjustment based on seed degrees**. First, the association score of a candidate with respect to the original seed set is computed. After generating a large number of random seed sets that represent the original set in terms of the degree distribution and size, association score of the candidate is computed using each of these random sets separately. Adjusted score of the candidate protein is then calculated as the statistical significance of the original association score, using this random population of association scores.

This statistical adjustment strategy is illustrated in Figure 3. Note that, since the multiple hypotheses being tested here are compared and ranked against each other (as opposed to accepting/rejecting individual hypotheses), it is not necessary to perform correction for multiple hypothesis testing.

### Reference model based on candidate degree

This reference model aims to assess the statistical significance of the association score of a protein *v *with respect to a population of association scores that belong to proteins with weighted degree similar to that of *v*. For protein *v*, if other proteins with similar weighted degrees usually have high association scores for a given seed set, the statistical significance of the association score of *v *can be considered low. On the other hand, loosely connected proteins are compared to other loosely connected proteins, which potentially have lower association scores, thus the artificial advantage of the highly connected proteins is removed.

This reference model is generated as follows:

• First, we compute the network-based association vector *α *with respect to the given seed set , again using Equation 1.

• Then, for each candidate gene , we select the *n *genes in the network with smallest *W *(*v*) *− W *(*u*) to create a representative set  that contains the *n *genes most similar to *v *in terms of their weighted degree (*n *= 1000 in our experiments).

• Subsequently, for each gene , we estimate the mean association score of its representative population as  and the standard deviation of association scores as .

• Finally, we compute the adjusted score of each candidate gene *v *as .

**Figure 4:**
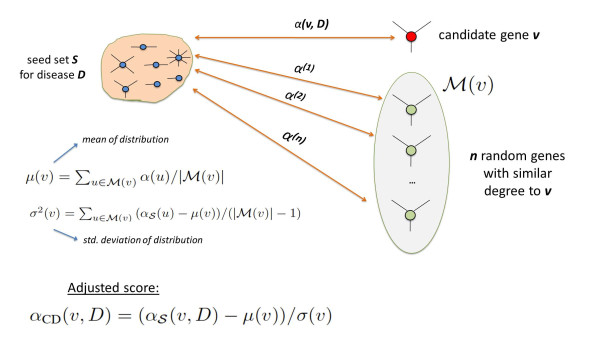
**Statistical adjustment based on candidate degree**. First, the association score of a candidate with respect to the original seed set is computed. Next, association scores of a large number of randomly selected proteins with similar degree to the candidate are computed using the original seed set. Adjusted score of the candidate protein is then calculated as the statistical significance of the original association score, using this random population of association scores.

In Figure 4, we illustrate this statistical adjustment strategy.

### Likelihood-ratio test using eigenvector centrality

Here, we assess the association of a gene with the seed set using a likelihood-ratio test. More precisely, considering *α*_RW_(*v, D*) as the likelihood of *v *being associated with the seed set  for disease *D*, we compare this likelihood with the likelihood of *v *being associated with any other gene product in the network. To compute the likelihood of *v*'s association with any other gene in the network, we use eigenvector centrality [23], which is precisely equal to the random walk based association score of *v *for zero restart probability (*r *= 0).

The objective here is to remove the bias introduced by network centrality, since central nodes are favored in the prioritization process by existing methods. The centrality score of a protein is computed again by simulating random walks, but this time with no restarts. More formally, the vector *α *that contains the association of each protein to the seed set is computed as in Equation 1 with restart probability *r >*0 (the selection of a particular value of *r *is discussed in detail in the Results section), while the centrality score of each protein is computed by setting *r *= 0. Indeed, setting *r *= 0 corresponds to the case where the seed set is empty, thereby making the resulting association score solely a function of the proteins's network centrality. For each , the eigenvector centrality based log-likelihood score is computed as:(3)

**Figure 5:**
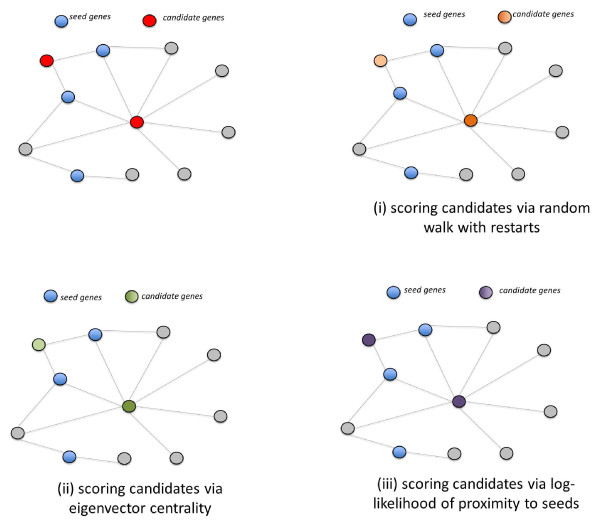
**Likelihood-ratio test using eigenvector centrality**. This statistical adjustment strategy is based on the eigenvector centrality of the candidate proteins. For the given sample network, seed proteins are represented by blue nodes and the intensity of the color of the candidates is proportional to their scores computed via different methods. In (*i*), two candidates are scored based on their proximity to seed proteins, calculated using random walk with restarts. In (*ii*), candidate proteins are scored based on their eigenvalue centrality in the network (without using any seed information). Finally in (*iii*), scores are assigned to candidates using the log-likelihood ratio of the values computed in (*i*) and (*ii*). Although the highly connected candidate (in the center of the network) is scored higher than the loosely connected candidate in (*i*) and (*ii*), the log-likelihood ratio of both candidates is similar as illustrated in (*iii*) since the association scores are adjusted by the centrality of the nodes in the network.

We illustrate these concepts on a small sample network in Figure 5, where we show the relative scores of two candidates computed using (*i*) network proximity with respect to seed proteins, (*ii*) eigenvector centrality and (*iii*) the log-likelihood ratio test.

### Uniform Prioritization

As we demonstrate in the next section, the statistical adjustment strategies presented above improve the performance of global prioritization algorithms in identifying loosely connected disease genes. However, this comes at the price of increased number of false negatives for highly connected disease genes. Motivated by this observation, we propose several uniform scoring strategies that aim to take advantage of both raw and statistically adjusted scores. The idea here is to derive a uniform prioritization method that uses the adjusted scores for loosely connected candidate genes, while using the raw scores for highly connected candidate genes.

For this purpose, we first sort the raw crosstalk scores (*α*_RW _or *α*_NP _) of candidate genes in descending order. Let *R*_RAW_(*v*) denote the rank of gene  in this ordering. Clearly, for , *R*_RAW_(*v*) *< R*_RAW_(*u*) indicates *v *is more likely to be associated with the disease than *u *is. Similarly, we sort the statistically adjusted scores (*α*_SD_, *α*_CD_, or *α*_EC_) in descending order, to obtain a rank *R*_ADJ_(*v*) for each gene .

We propose three alternate strategies for merging these two rankings to obtain a uniform ranking *R*_UNI_, where the objective is to have *R*_UNI_(*v*) *< R*_UNI_(*u*) if gene *v *is associated with the disease, while gene *u *is not. Once *R*_UNI_(*v*) is obtained using one of the following methods, we map it into the interval  in the obvious way.

### Uniform prioritization based on the degree of candidate gene

This uniform prioritization method chooses the ranking of each candidate gene based on its own weighted degree. Namely, for a given user-defined threshold *λ*, we define  as:(4)

for each . Thus the ranking of a highly connected gene is based on its raw association score, while that of a loosely connected gene is based on the statistical significance of its association score. Note that, with respect to this definition, the ranking of two genes can be identical, but there cannot be more than two genes with identical ranking. In the case of a tie, the tie is broken based on the unused ranking of each gene.

### Optimistic uniform prioritization

This approach uses the best available ranking for each candidate gene, based on the expectation that a true disease gene is more likely to show itself in at least one of the rankings as compared to a candidate gene that is not associated with the disease. Namely, we define  as:(5)

for each . Again, ties are broken based on the unused rankings.

### Uniform prioritization based on degree of known disease genes

Based on the notion that some diseases are studied more in detail compared to other diseases, we expect the degrees of genes associated with similar diseases to be somewhat close to each other. Statistical tests on disease associations currently available in the OMIM (Online Mendelian Inheritance in Man) database confirms this expectation (data not shown). We take advantage of this observation to approximate the network degree of the unknown disease gene in terms of the weighted degrees of the known disease genes. This enables having a global criterion for choosing the preferred ranking for all genes, as opposed to the gene-specific (or "local") criteria described above.

For a given seed set , we first compute . Subsequently, if  (where *λ *is defined as above), we set  for all , otherwise, we set . Observe that, this approach is global, *i.e*. rankings used do not depend on the weighted degree of each candidate gene, but the average weighted degree of the seed set. On the other hand, the other two uniform prioritization methods presented earlier are local, *i.e*. ranking used for each candidate gene depends on the gene being considered.

### Datasets

We test and compare the proposed methods on a comprehensive set of disease association data, using an integrated human PPI network in which interactions are associated with reliability scores. We describe these datasets in detail below.

### Disease Association and Phenotypic Similarity Data

We obtain disease information from the Online Mendelian Inheritance in Man (OMIM) database. OMIM provides a publicly accessible and comprehensive database of genotype-phenotype relationship in humans. We map genes associated with diseases to our PPI network and remove those diseases for which we are unable to map more than two associated genes. After this step, we have a total of 206 disease families with at least 3 associated genes. Number of genes associated with these diseases ranges from 3 to 36, with the average number of associations for each disease being approximately 6.

As mentioned previously, proteins associated with similar diseases or phenotypes lie in close proximity in the PPI network. This brings the idea of utilizing disease similarity information in to identification of disease genes [3,14]. Driel et al. [24] incorporate disease similarity information using a text mining algorithm that can be summarized as follows:

• First, each OMIM record is parsed and the keywords are searched for existence in the anatomy (A) and the disease (C) sections of the Medical Subject Headings Vocabulary (MeSH), which is a controlled vocabulary of U.S. National Library of Medicine. MeSH is especially useful for applications that use information that contains different terminology for identical concepts.

• Each OMIM record is then represented by binary vectors where each entry of the vector corresponds to the existence of a term in that record.

• Similarity of two diseases is then computed by calculating the cosine of the angle between their representative vectors.

After these calculations, a similarity score between 0 and 1 for every pair of OMIM diseases is available. This disease similarity information is used to compute the prior association between each gene and the disease of interest as follows: Let *d_c _*denote the disease of interest, *θ *= {*d*_1_, *d*_2_, *d*_3_, ..., *d_t_*} represent the set of other diseases for which information is available and *ϕ*(*d_i_, d_j _*) denote the similarity between diseases *d_i _*and *d_j_*. Also, let *S_i _*denote the set of genes associated with disease *d_i_*. Note that, these sets are not always disjoint, *i.e*. there are some genes that are associated with multiple diseases.

For the disease of interest *d_c_*, these methods consider other diseases *d_i _*in *θ *such that *ϕ*(*d_c_, d_i_*) *> γ *where *γ *is a user-defined threshold that is used to decide which diseases are considered similar. We follow [14] and utilize a logistic function to represent these similarity scores based on the empirical findings related to setting of *γ *in [24]. Now since vector *σ*(*v, D*) in Equations 1 and 2 represent the prior information on the association between each gene and the disease *d_c_*, *σ*(*v, D*) can be set to the similarity of the disease that involves gene *v *and the disease of interest *d_c_*.

Note that, if a gene is associated with more than one disease, its prior association score with *d_c _*is set to the maximum of the similarity scores of *d_c _*and the diseases it is associated with.

### Protein-Protein Interaction (PPI) Data

In our experiments, we use the human PPI data obtained from NCBI Entrez Gene Database [25]. This database integrates interaction data from several other databases available, such as HPRD, BioGrid, and BIND. After the removal of nodes with no interactions, the final PPI network contains 8959 proteins and 33528 distinct interactions among these proteins. Using a logistic regression model, we also assign reliability scores to each of these PPIs [26,27]. In this logistic regression model, we incorporate (i) the Pearson correlation of expression measurements across a range of different tissues and conditions for the corresponding coding genes (denoted *X*_1_(*uv*) for proteins *u *and *v*), (ii) the proteins' small world clustering coefficient (denoted *X*_2_(*uv*)) [28], and (iii) the proteins' subcellular localization data (denoted *X*_3_(*uv*)) [29,30]. For correlation of gene expression, the expression profiles of normal human tissues measured in the Human Body Index Transcriptional Profiling are used (GSE7307) [31]. In total, 213 normal samples are processed, representing over 90 distinct tissue types and a global expression correlation is acquired for each pair of interacting partners. The protein subcellular localization data is used to eliminate interactions that are not biologically relevant, based on the expectation that proteins that are not co-localized are not likely to interact with each other. Although proteins travel in the cell and can coexist in multiple compartments, incorporation of subcellular localization data helps eliminate many false negative interactions.

Once these statistics are obtained for each pair of proteins, we compute the reliability of interaction  as the probability of a true interaction between *u *and *v *given *X *(*uv*) = (*X*_1_(*uv*), *X*_2_(*uv*), *X*_3_(*uv*)), under the logistic distribution. Namely, we define , where *I*(*uv*) is the indicator random variable representing the existence of a true interaction between *u *and *v*. The parameters *β*_0_, *β*_1_, *β*_2_, and *β*_3 _are optimized to maximize the likelihood of a true interaction using training data that includes true positive, as well as true negative interactions. For this purose, we randomly select 1000 PPIs from the MIPS [32] database of interactions, an accepted gold standard for true positive interactions. The negative training set is acquired from randomly selected PPIs that are reported in Negatome, a database of proteins that are known not to interact with each other [33]. These experiments that involve random true positive and true negative interactions are repeated 1000 times and optimal values for all parameters are determined. Finally, the probability of each interaction, *i.e*., its reliability score, is calculated using these parameters.

### Experimental Setting

In order to evaluate the performance of different methods in terms of accurately prioritizing disease-associated genes, we apply leave-one-out cross-validation. For each gene that is associated with a disease, we conduct the following experiment:

• We remove that gene from the set of genes associated with the disease. We call the gene that is removed the *target gene *for that experiment. The remaining genes associated with the disease compose the seed set .

• We generate an artificial linkage interval, containing the target gene with other 99 genes located nearest in terms of genomic distance. The genes in this artificial linkage interval (including the target gene) compose the candidate set . Note that, according to our experiments, the size of candidate set does not have a significant effect on the performance difference between different methods as long as it is greater than 20 (data not shown).

• Using each of the methods described in the previous section, we obtain a ranking of candidate genes and use this ranking to predict disease genes.

In order to systematically compare the performance of different methods, we use the following evaluation criteria:

#### ROC curves

We plot ROC curves, *i.e*., *sensitivity vs*. 1-*specificity*, by thresholding the rank to be considered a "predicted disease gene" from 1 to 100. *Sensitivity *(recall) is defined as the percentage of true disease genes that are ranked above the particular threshold, whereas *specificity *is defined as the percentage of all genes that are ranked below the threshold. The area under ROC curve (AUC) is used as a measure to assess the performance of different methods. Note that, AUC is a conservative measure for this experimental set-up since there exists only one true positive (the target gene) for each experiment. For this reason, we also use other performance criteria that take into account the rank of the target gene.

#### Average rank

This is the average rank of the target gene among all candidate genes, computed across all disease-gene pairs in a total of ten executions of the experiments. Clearly, a lower number for average rank indicates better performance.

#### Percentage of the disease genes ranked in top 1% and 5%

Percentages of true disease genes that are ranked as one of the genes in the top 1% (practically, the top gene) and also in the top 5% among all candidates are also reported.

## Results and Discussion

In this section, we comprehensively evaluate the performance of the methods presented in the previous section. We start our discussion by investigating the effect of selection of the restart probability in random walk with restarts and network propagation algorithms. We then discuss in detail the effect of using the statistical adjustment methods. Next, we evaluate the performance of proposed uniform prioritization strategies and show that our final uniform method outperforms existing methods for network-based disease gene prioritization.

### Selection of Restart Probability

The restart probability represented by *r *in Equations 1 and 2 is a parameter that is used to adjust the preference between the importance of a protein with respect to the seed set and network topology. The effect of the selection of the restart probability is minor unless a very small value is used [34]. The performance degrades significantly for the values *r *≤ 0.01, which is expected because the effect of the seed genes is minimized in that case, thus the proximity of a protein is calculated based primarily on the centrality of that protein in the network. We observe that *r *= 0.3 is optimal for the performance of both random walk with restarts and network propagation algorithms, after running the algorithms with small increments of *r *[34]. Thus, *r *is set to 0.3 in all experiments presented in this paper, for all algorithms.

### Performance of Statistical Adjustment Methods

**Figure 6:**
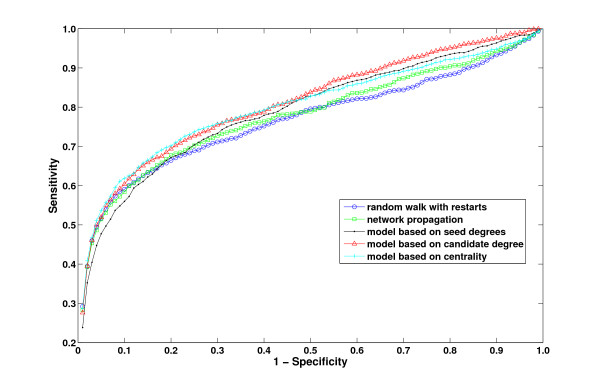
**ROC curves for the proposed statistical adjustment strategies and existing methods**.

As mentioned before, the performance of existing methods is highly biased with the connectedness of the true candidate protein. The effect of the total number of interactions of true disease gene on the performance of global methods is demonstrated in Figure 1. To investigate the effect of weighted degree on the proposed statistical adjustment strategies and existing methods, we compare the results achieved by different methods by considering the true disease genes with low weighted degree (*≤ m*) and with high weighted degree (*> m*) separately where  is the average weighted degree of all genes(*m *= 4.23 in our experiments). These results are shown in Table 1. As seen in the table, all of the three statistical adjustment methods outperform existing methods for loosely connected genes. On the other hand, existing approaches (raw scores) perform better for highly connected genes compared to the statistical adjustment methods. For this reason, when we consider the overall performance across all genes, the performance difference appears to be minor. However, as evident in the ROC curve in Figure 6, when all genes are considered, the statistical adjustment methods still perform better than existing methods. Next, we investigate how the proposed uniform prioritization methods improve the performance of these statistical adjustment methods.

**Table 1:** The effect of statistical adjustment on performance.

	All Genes		Degrees ≤ *m*		Degrees >*m*	
**Method**	**Avg. Rank**	**AUROC**	**Avg. Rank**	**AUROC**	**Avg. Rank**	**AUROC**

Network Propagation	22.75	0.77	30.00	0.70	15.57	0.84
Random walk w/restarts	23.75	0.76	34.76	0.65	12.82	0.88
Based on seed degree	21.42	0.78	21.51	0.79	21.35	0.79
Based on candidate degree	19.50	0.81	21.20	0.79	17.82	0.82
Based on centrality	20.40	0.80	24.35	0.75	16.47	0.84

The effect of statistical adjustment on performance. Average Rank of the true disease genes and AUC values are listed. To demonstrate the effect of connectivity, we also provide separate results for the cases in which the weighted degree of true disease gene is *≤ m *and *>m *where  is the average weighted degree of all genes (*m *= 4.23 in our experiments).

### Performance of Uniform Prioritization

Here, we systematically investigate the performance of the proposed uniform prioritization methods, by considering the combination of each of these methods with each of the three statistical adjustment methods (a total of nine combinations) applied in conjunction with random walk with restarts. In these experiments, the degree threshold *λ *is set to the average weighted degree of all genes. For convenience, we refer to each uniform prioritization method using the corresponding ranking symbol introduced in the previous section .

**Figure 7:**
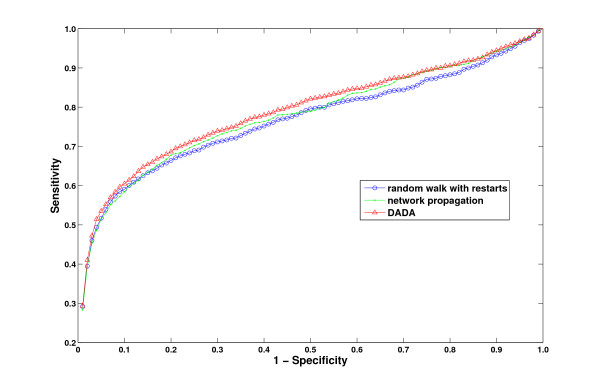
**ROC curves comparing the overall performance of DADA against existing methods**.

**Figure 8:**
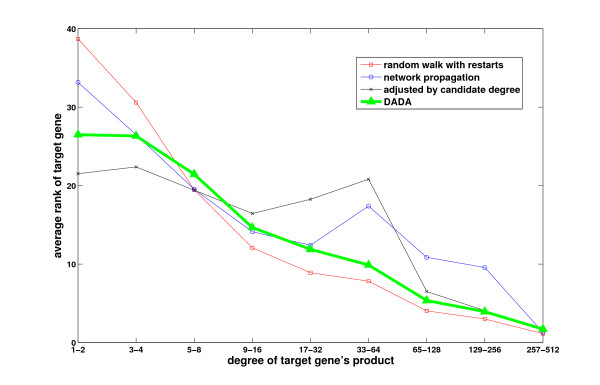
**The effect of connectivity of the target gene on overall performance of DADA**. Comparison of the performances of the proposed uniform prioritization method and existing methods with respect to the number of interactions of the target gene.

The average rank and AUC for the nine combinations of proposed methods are listed in Table 2. As seen in the table, it is difficult to choose between the proposed methods. We suggest that the uniform prioritization method based on seed degree , combined with statistical adjustment based on centrality, can be considered the "winner", since this approach is the only one that outperforms existing approaches in all performance metrics used. This setting is provided as the default combination of statistical adjustment and uniform prioritization methods in Matlab distribution of DADA. However, users are also given the option to configure these methods based on their needs, since the performance of these methods can be variable on different datasets. We compare this combination of proposed algorithms to existing global methods in Table 3 and Figure 7. These results reflect the best performance that can be achieved by DADA on the OMIM dataset. These results clearly show that DADA outperforms existing methods with respect to all performance criteria, however, the overall performance of DADA and Network Propagation are quite close to each other. Furthermore, careful inspection of average rank with respect to the number of interactions of true disease gene in Figure 8 shows that, this method almost matches the performance of the best performing algorithm for each degree regime. Namely, if the target gene is loosely connected, our uniform prioritization method performs similar to statistically adjusted version of the random walk with restarts algorithm. On the other hand, it performs similar to the original random walk with restarts algorithm (with raw scores) for highly connected target genes.

**Table 2:** Performance of all combinations of uniform prioritization methods.

	**Candidate deg**.			**Seed deg**.			Centrality		
									

Avg. Rank	20.89	20.82	20.56	21.07	21.10	21.38	21.14	20.95	21.52
AUROC	0.80	0.80	0.80	0.79	0.79	0.79	0.79	0.79	0.79
Perc. ranked in top 1%	28.18	26.70	28.01	24.63	24.07	26.18	27.85	27.93	29.41
Perc. ranked in top 5%	52.67	52.67	52.01	51.77	51.93	50.92	53.16	52.91	53.57

**Table 3:** Comparison of the proposed method with existing approaches.

METHOD	Avg. Rank	AUROC	Perc. Ranked in top 1%	Perc. Ranked in top 5%
DADA	21.52	0.79	29.41	53.57
Network propagation	22.75	0.77	28.18	51.52
Random walk w/restarts	23.75	0.76	29.25	51.76

### Case Example

**Figure 9:**
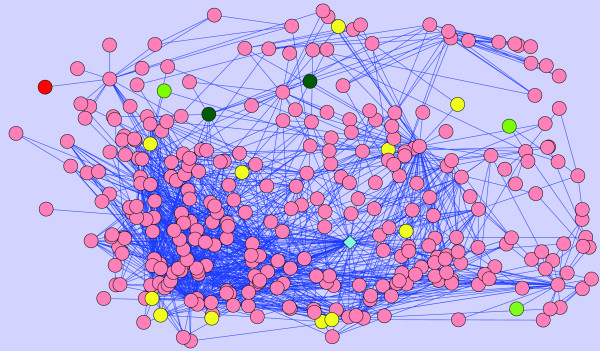
**Case Example**. Case example for the Microphthalmia disease. Products of genes associated with Microphthalmia or a similar disease are shown by green circles, where the intensity of green is proportional to the degree of similarity. The target disease gene that is left out in the experiment and correctly ranked first by our algorithm is represented by a red circle. The gene that is incorrectly ranked first for both of the existing global approaches is shown by a diamond. Other candidate genes that are prioritized are shown by yellow circles.

Here, we provide a real example to demonstrate the power of the proposed method in identifying loosely connected disease genes. We focus on *Microphthalmia *since it provides a clear example to illustrate the power of statistical adjustment in detecting true disease genes with few known interactions. *Microphthalmia *has 3 genes directly associated with it in the PPI network, namely *SIX6*, *CHX10 *and *BCOR*. In our experiments, we remove *SIX6 *and try to predict this gene using the other two genes, as well genes associated with diseases similar to Microphthalmia. This experiment is illustrated in Figure 9. The figure shows the 2-neighborhood of proteins *SIX6*, *CHX10 *and *BCOR*. As seen in the figure, the global methods fail because the product of *SIX6 *is not a centralized protein with a degree of only 1. Thus, random walk with restarts model ranks this true gene as 26*^th ^*and network propagation ranks it 16*^th ^*among 100 candidates. On the other hand, our method is able to correctly rank this gene as the 1*^st ^*candidate. Both random walk and network propagation rank the gene *AKT1 *top among all candidates, which, not surprisingly, is a high degree protein (78), also connected to other hub proteins.

## Conclusions

In this paper, we have shown that approaches based on global network properties in prioritizing disease-associated genes are highly biased by the degree of the candidate gene, thus perform poorly in detecting loosely connected disease genes. We proposed several statistical adjustment strategies that improve the performance, particularly in identifying loosely connected disease genes. We have shown that, when these adjustment methods are used together with existing global methods, the resulting method outperforms existing approaches significantly. These results clearly demonstrate that, in order to avoid exacerbation of ascertainment bias and propagation of noise, network-based biological inference methods have to be supported by statistical models that take into account the degree distribution. DADA is freely available for download at http://compbio.case.edu/dada/.

## Authors' contributions

RME conceived and formulated the problem; SE and MK conceived and formulated the proposed approach and algorithms; SE implemented and tested the algorithms, GB constructed the integrated PPI network; SE and MK drafted the manuscript; all authors reviewed, edited, and approved the final manuscript.
